# RBM3 and CIRP expressions in targeted temperature management treated cardiac arrest patients—A prospective single center study

**DOI:** 10.1371/journal.pone.0226005

**Published:** 2019-12-10

**Authors:** Lisa-Maria Rosenthal, Christoph Leithner, Giang Tong, Kaspar Josche Streitberger, Jana Krech, Christian Storm, Katharina Rose Luise Schmitt

**Affiliations:** 1 Dept. for Congenital Heart Disease/Pediatric Cardiology, Deutsches Herzzentrum Berlin, Berlin, Germany; 2 Berlin Institute of Health, Berlin, Germany; 3 Dept. of Neurology, Charité Universtitätsmedizin Berlin, Berlin, Germany; 4 Dept. of Internal Medicine, Nephrology and Intensive Care, Charité Universitätsmedizin Berlin, Berlin, Germany; 5 Dept. for Pediatric Cardiology, Charité Universitätsmedizin Berlin, Berlin, Germany; 6 DHZK (German Centre for Cardiovascular Research), Berlin, Germany; Azienda Ospedaliero Universitaria Careggi, ITALY

## Abstract

**Background:**

Management of cardiac arrest patients includes active body temperature control and strict prevention of fever to avoid further neurological damage. Cold-shock proteins RNA-binding motif 3 (RBM3) and cold inducible RNA-binding protein (CIRP) expressions are induced *in vitro* in response to hypothermia and play a key role in hypothermia-induced neuroprotection.

**Objective:**

To measure gene expressions of RBM3, CIRP, and inflammatory biomarkers in whole blood samples from targeted temperature management (TTM)-treated post-cardiac arrest patients for the potential application as clinical biomarkers for the efficacy of TTM treatment.

**Methods:**

A prospective single center trial with the inclusion of 22 cardiac arrest patients who were treated with TTM (33°C for 24 hours) after ROSC was performed. RBM3, CIRP, interleukin 6 (IL-6), monocyte chemotactic protein 1 (MCP-1), and inducible nitric oxide synthase (iNOS) mRNA expressions were quantified by RT-qPCR. Serum RBM3 protein concentration was quantified using an enzyme-linked immunosorbent assay (ELISA).

**Results:**

RBM3 mRNA expression was significantly induced in post-cardiac arrest patients in response to TTM. RBM3 mRNA was increased 2.2-fold compared to before TTM. A similar expression kinetic of 1.4-fold increase was observed for CIRP mRNA, but did not reached significancy. Serum RBM3 protein was not increased in response to TTM. IL-6 and MCP-1 expression peaked after ROSC and then significantly decreased. iNOS expression was significantly increased 24h after return of spontaneous circulation (ROSC) and TTM.

**Conclusions:**

RBM3 is temperature regulated in patients treated with TTM after CA and ROSC. RBM3 is a possible biomarker candidate to ensure the efficacy of TTM treatment in post-cardiac arrest patients and its pharmacological induction could be a potential future intervention strategy that warrants further research.

## Introduction

Cardiac arrest (CA) is associated with high morbidity and mortality, and imposes a significant burden on the healthcare system [1]. Although cardiovascular failure is usually the main cause of early mortality after CA, the majority of late deaths are a result of active termination of life support after a prognosis of poor neurological outcome [2]. Experimental and clinical data indicate that targeted temperature management (TTM) is neuroprotective after global cerebral hypoxia-ischemia by modulating various cellular pathways, reducing oxygen consumption, and impairing the release of cytotoxic agents, as well as delaying cell death [3, 4]. Whereas previously published trials showed a benefit of hypothermia (32–34°C for 24 hours) compared to normothermia in patients with out-of-hospital cardiac arrest (OHCA), no significant differences in the combined death or poor neurological functional outcome was observed between 33 versus 36 °C in the TTM trial [5–7].

Global protein synthesis and cell metabolism are generally suppressed when body temperature is decreased. Contrarily, a small subset of cold-responsive proteins is induced, including RNA-binding motif 3 (RBM3) and cold-inducible RNA-binding protein (CIRP). [8] Both proteins are ubiquitously expressed in various cell types and share a high amino acid sequence similarity with a conserved RNA-recognition motif, which enables them to bind RNA [8, 9]. Interestingly, exposure to 36 °C is sufficient to significantly induce RBM3 expression *in vitro* [10]. However, both CIRP and RBM3 reach their peak expression at mild-to-moderate hypothermia (28–34 °C), whereas hyperthermia (39–42 °C) significantly decreases their expression [8, 9, 11]. Furthermore, endogenous and environmental stressors including hypoxia and radiation have been demonstrated to affect RBM3 and CIRP expressions [12–14].

The cellular functions and biological activities of RBM3 and CIRP appear to be numerous and remain largely unknown. Both RBM3 and CIRP have the capacity to bind RNA and seem to play a key role in post-transcriptional RNA modulation and translation in order to enhance global protein synthesis under stressful cellular conditions [15]. They are involved in cell proliferation, promotion of cell cycle progression, and impairment of apoptosis [16–18]. *In vitro* data indicates that RBM3 mediates hypothermia-induced neuroprotection, although the underlying mechanism remains to be elucidated [19]. Notably, RBM3 induction prevents neuronal cell death and promotes synapse reassembly in a mouse model of Alzheimer’s and prion diseases, thus delaying the progression of chronic neurodegeneration [20]. The role of CIRP in hypoxic-ischemic brain injury remains controversial. Whereas overexpression of CIRP reduces H_2_O_2_-induced apoptosis, indicating a neuroprotective role, secretion of CIRP by microglia after cerebral ischemia leads to TNF-*α* and IL-1*β* mediated neuroinflammation and increases neuronal cell death [18, 21–23]. Extracellular CIRP has been identified as an inflammatory mediator in sepsis and is associated with a poor prognosis [24, 25]. However, the role of both cold-shock proteins as potential biomarkers for the efficacy of targeted temperature management (TTM) has yet to be investigated. Therefore, we aim to investigate the possibility of measuring RBM3 and CIRP expressions, as well as their regulation and those of the inflammatory biomarkers interleukin 6 (IL-6), monocyte chemotactic protein 1 (MCP-1), and inducible nitric oxide synthase (iNOS) in patients treated with TTM after CA.

## Methods

### Study design

A single-center prospective trial was performed at the Circulatory Arrest Center at Charité Universitätsmedizin Berlin, Germany during the period of 02/2016-05/2017. The study was approved by the institutional ethics committee of Charité Universitaetsmedizin Berlin, Berlin, Germany (decision EA4/024/16) and a written consent was obtained from a legal custodian. Patients older than 18 years suffering from cardiac arrest (OHCA and IHCA), requiring CPR, successfully regaining ROSC, and receiving targeted temperature management were enrolled. Post-resuscitation care was provided according to institutional standard procedures and current guidelines. [26] TTM was conducted with a target temperature of 33 °C that was maintained for 24 hours. Patients were subsequently rewarmed at a rate of 0.25 °C per hour until reaching a body temperature of 36 °C. Serum samples were collected from the central venous line upon admission to the ICU and 24, 48, and 72 hours later. Neurological outcome was assessed using the cerebral performance category score (CPC), ranging from 1 (no or minor neurological deficits) to 5 (death), and neuron specific enolase (NSE) levels in blood at 72 hours after admission to the ICU. Good neurological outcome was defined as CPC 1–3 and poor outcome as CPC 4–5.

### RNA isolation and real-time quantitative PCR

Total RNA was isolated from whole blood using the Tempus^™^ Blood RNA Tube (Foster City, CA, USA) and Tempus^™^ Spin RNA Isolation Reagent Kit (Warrington, UK) according to established manufacturers’ protocols (https://dx.doi.org/10.17504/protocols.io.2vwge7e). Total RNA concentration and purity were analyzed by spectrophotometric determination at 260 nm and 280 nm using a NanoDrop-2000 UV-Vis spectrophotometer (Thermo Fisher Scientific, DE, USA). Prior to reverse transcription, genomic DNA contamination was ruled out by running RNA samples on a 1% agarose gel and visualized with ethidium bromide (dx.doi.org/10.17504/protocols.io.2v5ge86). Subsequently, 2 μg of total RNA was reverse transcribed to single-stranded cDNA in 20 μl of reaction mixture with RNase inhibitor using the High Capacity cDNA Reverse Transcription Kit (Applied Biosystems, Darmstadt, Germany) according to manufacturer’s protocol in a MJ Research PTC-200 Thermal Cycler (MJ Research Inc., St. Bruno, Canada). (https://dx.doi.org/10.17504/protocols.io.2v6ge9e) Expression of target genes and endogenous control, GAPDH, mRNA was assessed by real-time quantitative PCR using the TaqMan^®^ Gene Expression Assays on a StepOnePlus^™^ Real-Time PCR System (Applied Biosystems, Darmstadt, Germany) according to the manufacturer’s recommendations. (dx.doi.org/10.17504/protocols.io.2v8ge9w) The ΔC_t_ method was used to determine expression levels for each gene of interest by normalizing the quantified mRNA to GAPDH. Relative gene expression was normalized to pre-cooling values at ROSC using the ΔΔC_t_ method and the RQ values were used to test for significant differences between observed time points. Gene expression assays (Applied Biosystems, Darmstadt, Germany) that were used are listed in Table 1.

**Table 1:** Gene expression assays.

Gene	Gene Assay ID	Gene	Gene Assay ID
RBM3	Hs00943160_g1	CIRP	Hs00989762_g1
IL-6	Hs00174131_m1	MCP-1 (Ccl2)	Hs00234140_m1
iNOS	Hs01075529_m1	GAPDH	Hs02786624_g1

### Enzyme-Linked Immunosorbent Assay (ELISA) for RBM3 protein analysis

RBM3 protein in serum samples was assessed using the Human RBM3 ELISA Kit (BioTeZ Berlin-Buch GmbH, Berlin, Germany). Samples were prepared according to the manufacturer’s instructions and analyzed using a Multiskan Ascent Plate Reader (MTX Lab Systems, LLC, Bradenton, FL, U.S.A.). The concentration of RBM3 in serum samples was interpolated from a standard curve. (http://dx.doi.org/10.17504/protocols.io.3p5gmq6).

### Neuron specific enolase (NSE) measurement

NSE serum concentration was determined in clinical routine 3 days after CA by our institutions laboratory (Labor Berlin) using an ELISA (Roche *Cobas*, Roche Diagnostics, Basel, Switzerland).

### Statistical analysis

Data were graphed and analyzed using Prism GraphPad 7 (GraphPad Software Inc., La Jolla, CA, USA). Variables are expressed as mean +/- standard error, median with interquartile range (IQR), or percentages as appropriate. Difference between non-parametric parameters was tested using a Mann-Whitney U test. Difference between non-parametric groups was compared using a Kruskal-Wallis one-way analysis of variance. Grouped analyses were corrected for multiple comparisons using the Dunn method with alpha = 0.05. P<0.05 was considered statistically significant.

## Results

### Patients and outcome

**Fig 1:**
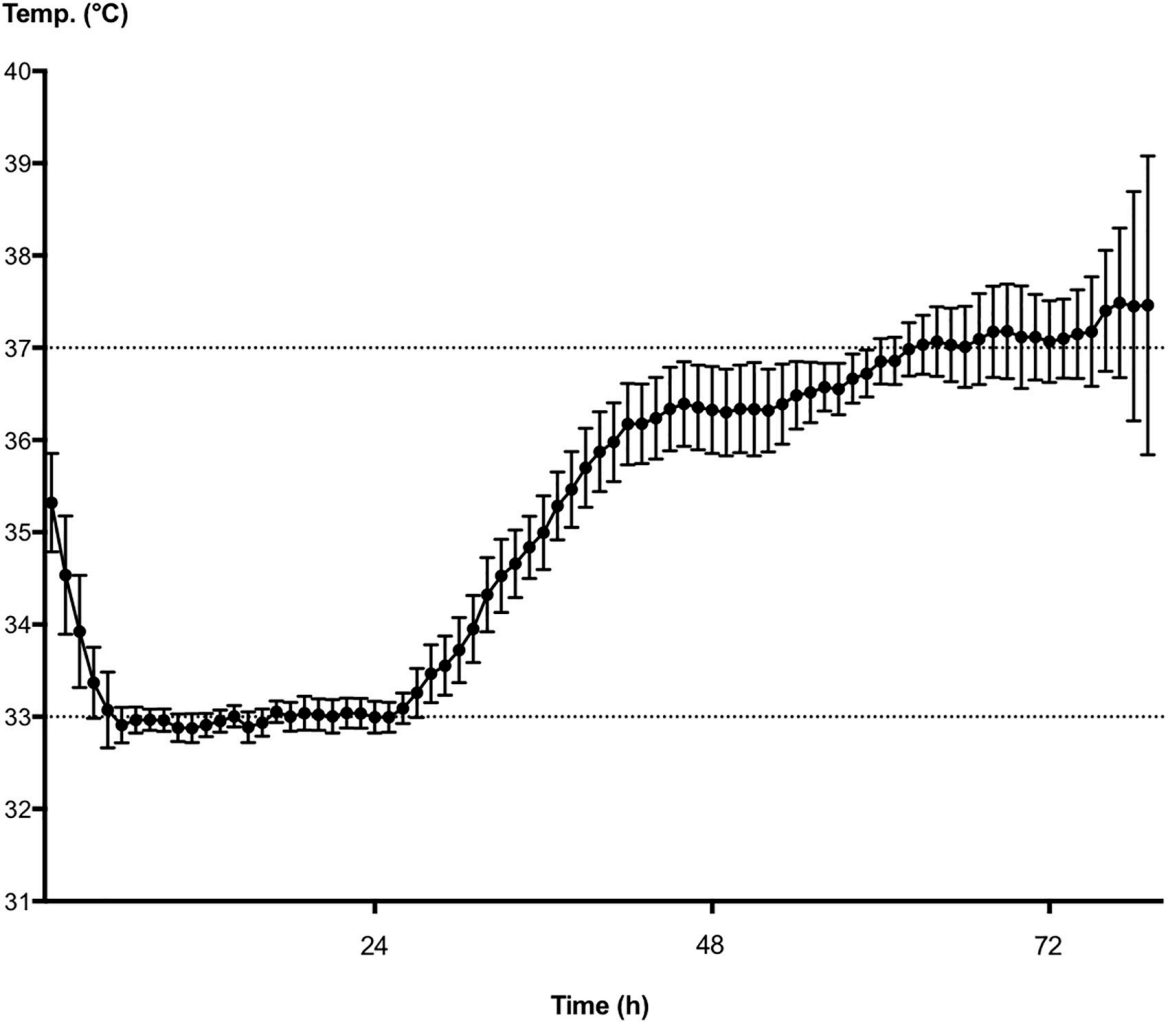
Body temperature of patients during the examination period. (n = 22, mean +/- SD).

**Fig 2:**
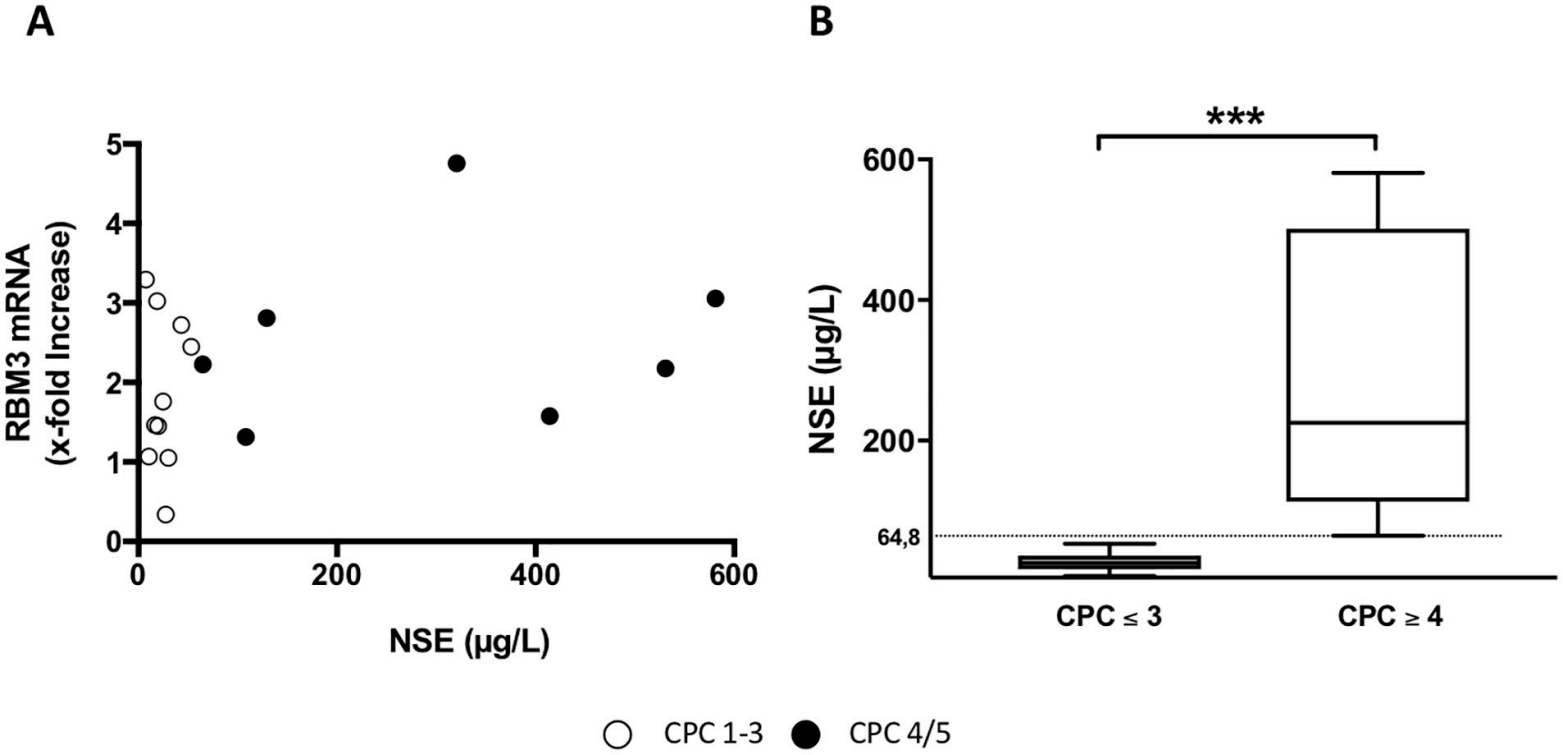
**A)** RBM3 mRNA increase in correlation with NSE levels **B)** NSE in correlation with CPC. Patients with a CPC 1, 2 or 3 are marked with white points and patients with a CPC 4 or 5 with black points. (mean +/- SD, *** p < 0,001).

22 patients met the inclusion criteria and patients’ characteristics were summarized in Table 2. The majority of patients were male (91%) with a median age of 67 years (IQR 56–73), had an out-of-hospital cardiac arrest (82%), and primary arrest etiology was cardiac (73%) followed by respiratory causes (9%). The median time to return of spontaneous circulation (tROSC) was 20 minutes (IQR 11–30) and was longer in patients with OHCA than with IHCA. TTM was maintained for a median time of 25 hours (IQR 24–29) with a targeted temperature of 33°C (Table 3 and Fig 1). The overall neurological outcome was acceptable with a median best CPC Score of 2 (IQR 2–5, Table 3). Seven patients (32%) died or had a poor neurological outcome (CPC 4 or 5, Table 3). Of those who survived to discharge (14 patients, 64%) the majority (12 patients, 55%) had a good neurological outcome (CPC 1–3, Table 3). Median NSE level 72 hours after ROSC was 37 μg/ml (IQR 19.7–129, Table 3). NSE levels were significantly higher in patients with CPC 4 or 5, and considered as poor neurological outcome (Fig 2B).

**Table 2:** Cardiac arrest characteristics. Baseline characteristics are displayed as absolute numbers and percentages or median with interquartile range (IQR).

**Demographic data**	
Patients	22
Male, n (%)	20 (91)
Female, n (%)	2 (9)
Age,y, median (IQR)	67 (56–73)
**Cardiac arrest**	
Out-of-hospital (OHCA), n (%)	18 (82)
In-hospital (IHCA), n (%)	4 (18)
tROSC, min, median (IQR)	20 (11–30)
IHCA, min, median (IQR)	10 (4–26)
OHCA, min, median (IQR)	23 (14–32)
Bystander CPR applied	7 (39)
Bystander CPR not applied	4 (22)
unknown	7 (39)
Initial rhythm shockable	12 (55)
Initial rhythm non-shockable	10 (45)
**Primary arrest etiology**	
Cardiac, n (%)	16 (73)
Respiratory, n (%)	2 (9)
Neurological, n (%)	1 (5)
Other/unknown, n (%)	3 (14)

**Table 3:** Targeted temperature management (TTM) and outcome. Baseline characteristics are displayed as absolute numbers and percentages or median with interquartile range (IQR).

**Targeted temperature management**	
Target temperature (°C)	33°C
Duration, h, median (IQR)	25 (24–29)
Best Cerebral Performance (CPC) Score	2 (2–5)
CPC 1, n (%)	1 (5)
CPC 2, n (%)	11 (50)
CPC 3, n (%)	2 (9)
CPC 4, n (%)	1 (5)
CPC 5, n (%)	7 (32)
Neuron specific enolase 72 hrs after ROSC (μg/L), median (IQR)	37 (19,7–129)
**Survival**	
Survival to discharge, n (%)	14 (64)
Death, n (%)	8 (36)
early, CA-related	7 (32)
late, other causes	1 (5)

### RBM3 and CIRP mRNA expression

**Fig 3:**
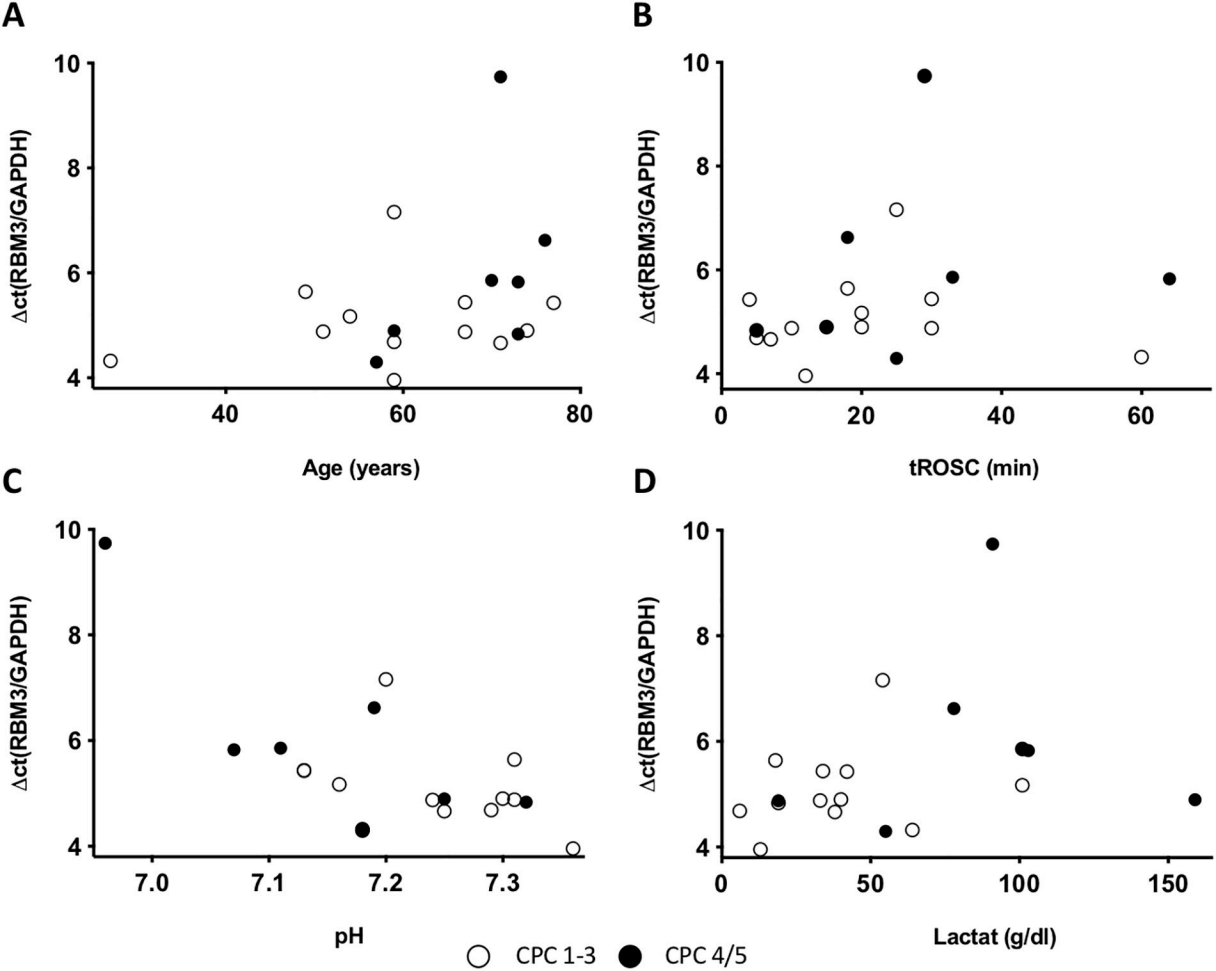
Baseline RBM3 mRNA expression in correlation with A) the patients age, B) tROSC, C) pH, and D) lactate in the initial blood gas analysis. Patients with a CPC 1, 2, or 3 are marked with white points and patients with a CPC 4 or 5 with black points.

**Fig 4:**
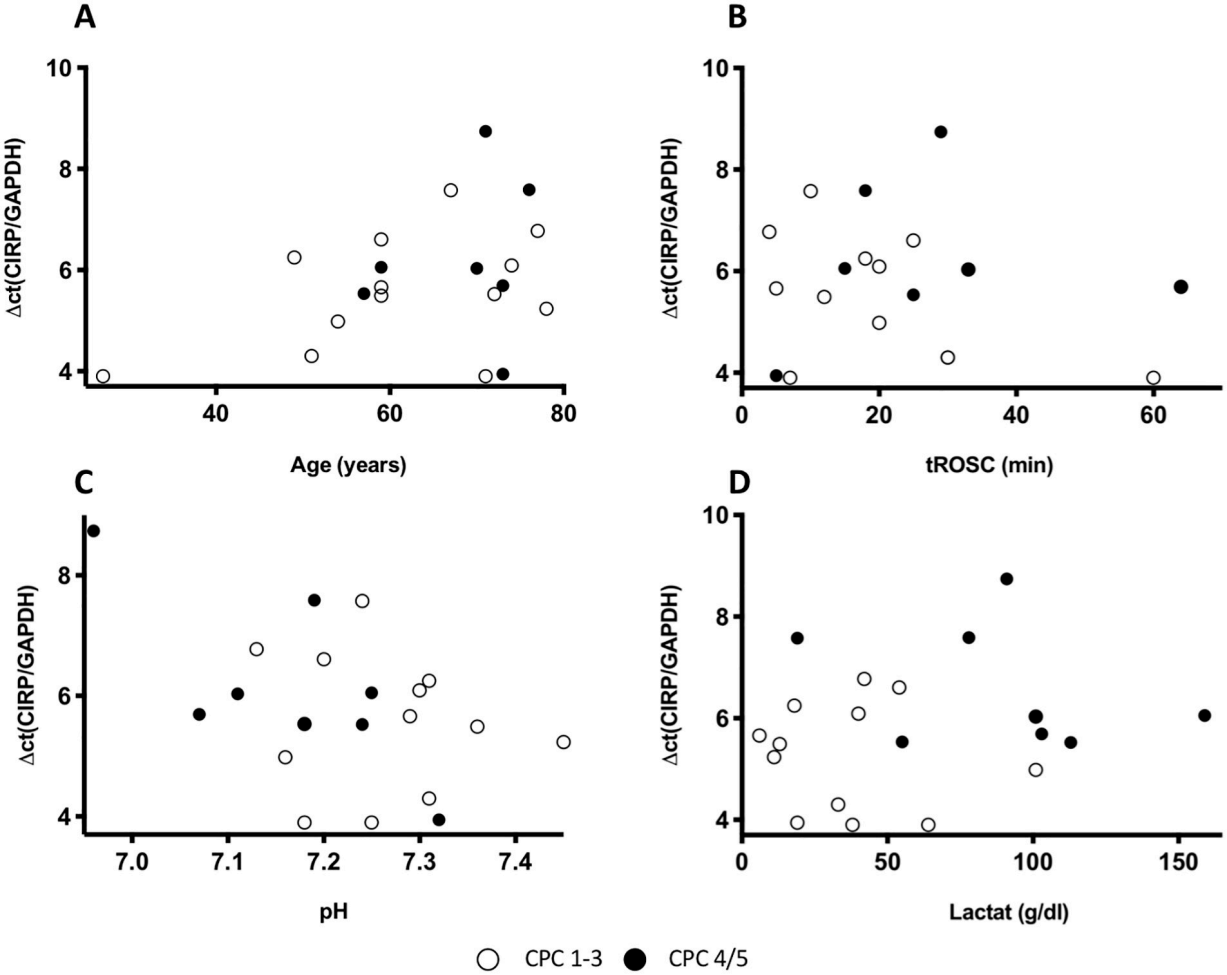
Baseline CIRP mRNA expression in correlation with A) the patients age, B) tROSC, C) pH, and D) lactate in the initial blood gas analysis. Patients with a CPC 1, 2, or 3 are marked with white points and patients with a CPC 4 or 5 with black points.

**Fig 5:**
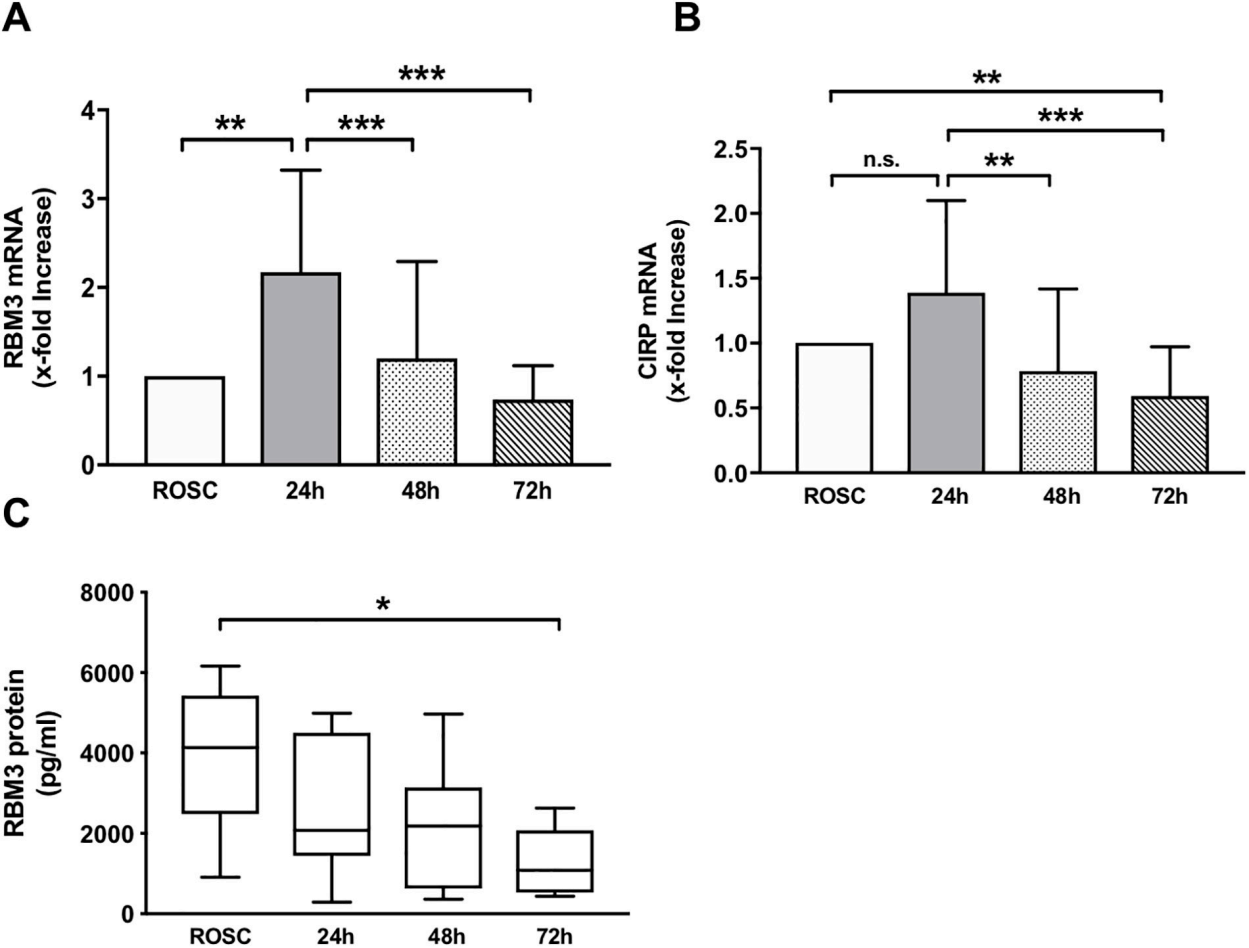
Expression of cold-responsive proteins A) RBM3 mRNA, B) CIRP mRNA, and C) RBM3 serum protein concentration. Data are shown as mean +/- SD and * p < 0.05, ** p < 0.01, and *** p < 0.001 were considered significant.

The baseline mRNA expression of RBM3 and CIRP after admission to the ICU was heterogenous and did not correlate with the patients’ age, the time to return of spontaneous circulation (tROSC) or the extent of hypoxia as reflected by levels of lactate and pH in the initial blood gas analysis (Figs 3 and 4, respectively). RBM3 mRNA expression significantly increased by 2.2-fold in response to TTM (Fig 5A). After rewarming RBM3 mRNA expression significantly decreased in a time-dependent manner and returned to baseline levels after 72 h. A similar mRNA expression kinetic was also observed for CIRP with an increase of 1.4-fold in response to mild hypothermia and decrease after rewarming without reaching statistical significance (Fig 5B). CIRP mRNA expression after 72 h was significantly lower than baseline CIRP before cooling. No correlation between RBM3 mRNA x-fold increase and NSE concentration was observed (Fig 2A).

### RBM3 protein serum concentration

Unlike mRNA expression RBM3 protein serum concentration did not increase in response to TTM (Fig 5C). The highest RBM3 protein concentration (mean 3895 pg/ml) was measured immediately after ROSC, slowly decreased during the observation time (mean 2642 pg/ml at 24 h and 2076 pg/ml at 48 h), and was significantly lower after 72 h (mean 1293 pg/ml) as compared to the pre-cooling sample.

### Expression of inflammatory cytokines IL-6, iNOS, and MCP-1

**Fig 6:**
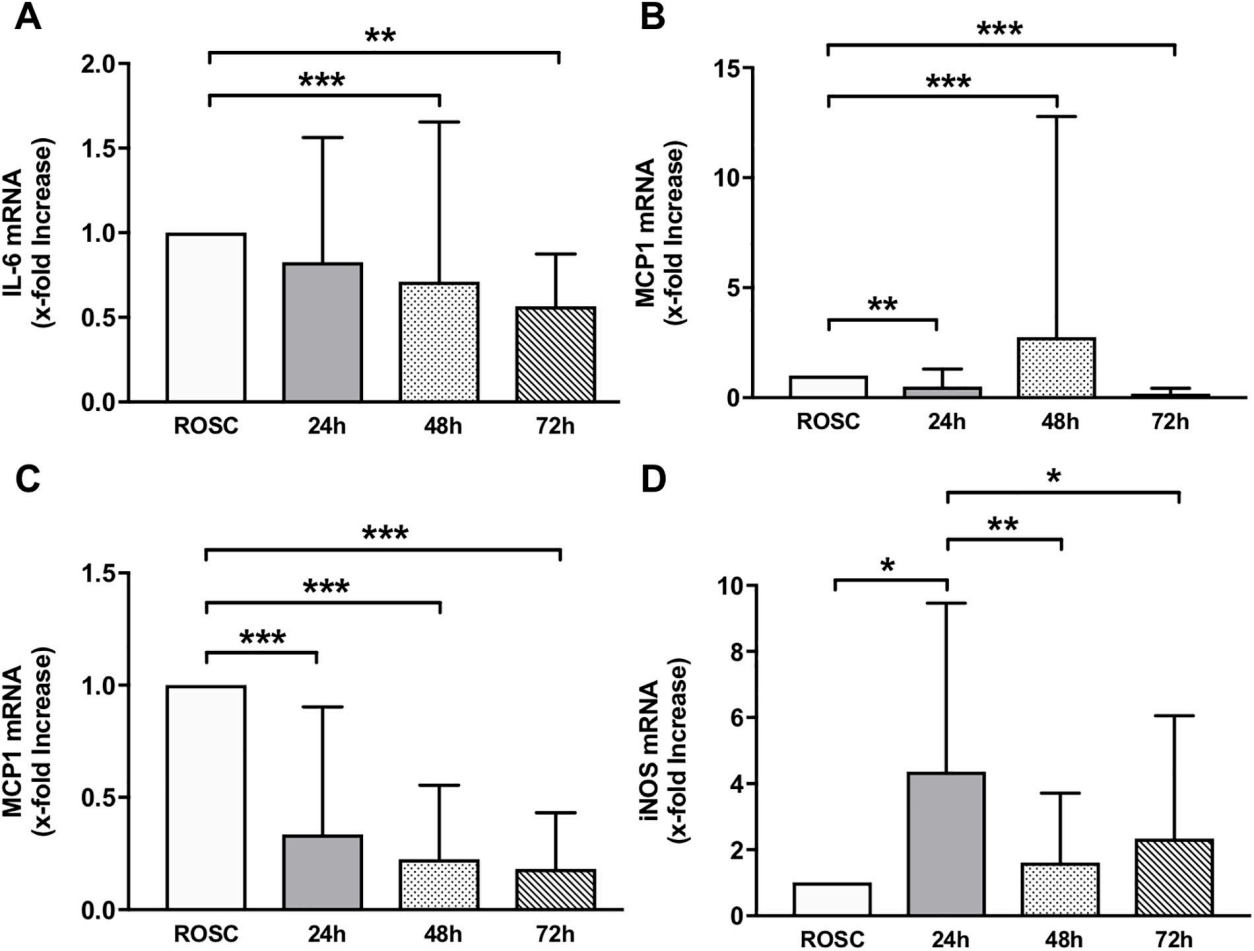
Expression of *inflammatory cytokines* A) IL-6, B) MCP-1 for all patients, C) MCP-1 excluding the two patients with high MCP-1 at 48 h who died shortly afterwards, and D) iNOS mRNA x-fold increase. Data are shown as mean +/- SD and * p < 0.05, ** p < 0.01, and *** p < 0.001 were considered significant.

Interleukin-6 (IL-6) mRNA expression peaked after CA and ROSC and then significantly decreased after rewarming at 48 and 72 h (Fig 6A). Similar kinetics were observed with MCP-1 mRNA expression, which was highest after CA and ROSC and then significantly decreased in a time dependent manner. Interestingly, two patients had highly increased MCP-1 mRNA expression at 48 h and died shortly afterwards. Data for all patients is illustrated in Fig 6B and without the two deceased patients in Fig 6C. iNOS mRNA expression was significantly increased 24 h after ROSC, as well as after TTM, and then significantly decreased again (Fig 6D).

## Discussion

In the present study, which is the first to investigate the temperature-dependent regulation of cold-shock proteins in patients after cardiac arrest, we demonstrate that RBM3 mRNA expression is significantly induced by TTM in post-cardiac arrest patients. A similar mRNA expression kinetic was also observed for CIRP without reaching statistical significance.

### RBM3 mRNA and protein expression

Experimental data suggests a key role for the RBM3 cold-shock protein in hypothermia-induced neuroprotection [19]. To date, all research pertaining to the regulation and function of RBM3 has been conducted *in vitro* or in small animal models. With the present study we are the first to our knowledge to observe an upregulation of RBM3 mRNA expression in blood samples of post-cardiac arrest patients treated with TTM (Fig 5A). The increase in RBM3 mRNA expression was most likely induced by hypothermia as other possible confounders including age, degree of hypoxia (pH, lactate), or tROSC had no observable effect (Fig 3). Our findings are in correlation with previously published data originating from cell or tissue culture models [8, 11, 13]. Previously published trials have correlated elevated NSE concentration with poor neurological outcome (Fig 2B) [27]. No correlation between RBM3 mRNA expression and NSE serum levels could be observed in our study (Fig 2A). Therefore, no conclusion about the correlation of RBM3 concentration and the neurological outcome of the rescued patients could be drawn from our small patient cohort.

The majority of previously published RBM3 expression data was observed in young animals and primary cells. A dynamic regulation of RBM3 expression with regard to age has been observed, showing high expression levels during the early phase of brain development followed by a decline over time in most brain regions [19, 28]. Data regarding the neuroprotective significance of RBM3 in adult animals is almost entirely lacking [29]. Our novel findings that RBM3 mRNA expression is temperature-regulated in an elderly patient cohort indicates that even adult patients could benefit from TTM-induced RBM3 neuroprotection.

We further investigated if the increase in TTM-induced RBM3 mRNA expression is translated to RBM3 protein expression. To our knowledge, we are the first to report measurable serum RBM3 protein concentrations in a human post CA cohort. However, no corresponding increase in RBM3 protein in response to TTM was observed in our patient cohort. This is probably explained by the assumption that RBM3 is predominantly located intracellular and no active secretion mechinism has been reported. Therefore, RBM3 is not known to be actively release into the circulatory system and any changes in protein expression would not be measurable in the serum. At a subcellular level RBM3 has been shown to be localized primarily to the cell nucleus to better execute its various biological functions, including post-transcriptional modulation of RNA-stability and translation, regulation of cell cycle progression, promotion of cell proliferation, and inhibition of apoptosis [15, 16, 18, 30]. To date, the question if RBM3 protein is actively secreted from the cells and has extracellular functions remains unknown and warrants further research. Notably, RBM3 protein concentration in our study cohort decreased over the observational period and was significantly lower after 72 h as compared to the pre-cooling sample. The high measurable RBM3 protein levels after CA and ROSC could possibly be the result of necrotic cell death resulting from CA-induced ischemic injury. Moreover, the measurable decrease in serum RBM3 protein following rewarming can be attributed to reestablishment of a functional circulation and fluid volume management on the ICU.

### CIRP mRNA expression

In contrast to RBM3 the role of CIRP in neuroprotection is less clear and remains controversial. Previously published data indicate that CIRP is also involved in hypothermia-induced neuroprotection, but also triggers inflammatory responses in hemorrhagic shock and sepsis patients [18, 21, 24, 25]. In our study cohort CIRP mRNA expression showed a similar expression kinetic to RBM3 with induction in response to TTM, but did not reach statistical significance. These results correlate with previously published data on hypothermia-induced CIRP expression in experimental studies [8, 11]. Similar to RBM3, this regulation in mRNA expression was not confounded by the patients’ age, extent of hypoxia, or tROSC. Notably, CIRP expression was significantly decreased at 72 hours after ROSC as compared to pre-cooling, suggesting that the CIRP baseline level was increased possibly as a result of ischemia-induced necrosis and in response to cellular stressors during CA and resuscitation attempts. However, this is speculative as we did not have a “healthy control” group of patients before CA. The milder temperature-dependent regulation of CIRP in comparison to RBM3 in our patient cohort could be explained by its multifarious biological activities. Systemic inflammatory responses are common in post-cardiac arrest patients and therefore, could possibly have an influence on CIRP mRNA expression [31].

### Inflammatory response

Post-cardiac arrest patients present with sepsis-like systemic inflammation and impaired vasoregulation due to endothelial dysfunction [32]. We observed high mRNA expressions of the proinflammatory cytokines IL-6 and MCP-1 after CA and ROSC, which were significantly attenuated after TTM. If this decrease was indeed mediated by TTM, as previously seen in a post-ischemic rat heart model, remains unclear due to a missing normothermic control group [33]. Increased IL-6 expression has been recently reported to correlate with a poor neurological outcome in post-cardiac arrest patients [32]. However, we observed no correlation between IL-6 expression and neurological outcome in our study cohort, possibly due to the limited cohort size. Notably, MCP-1 expression was strongly increased in two patients at 48 h, who died shortly afterwards. High levels of MCP-1 protein have been reported in critically ill patients with sepsis [34]. However, the role of MCP-1 in post cardiac arrest patients has not been investigated. Our data suggests that MCP-1 mRNA expression could be a relevant predictor in critically ill patients with systemic inflammation after CA and therefore, warrants further investigation.

Additionally, iNOS mRNA was observed to be highly expressed 24 h after ROSC at the end of the TTM phase. This induction may be attributed to either a delayed response to the ischemia/reperfusion-induced injury or to the hypothermic treatment itself. Increased iNOS activity after CA/ROSC has been reported to lead to the production of large quantities of toxic NO, resulting in contractile dysfunction corresponding with a decreased systolic and diastolic cardiac function [35]. Contrarily, inhibition of iNOS expression prior to CA and resuscitation has been shown to protect myocardial function after ROSC [36]. TTM has been reported to attenuate iNOS protein production in the post-ischemic rat hearts, which we did not observe in our patient cohort [33]. Further research is required to elucidate the clinical relevance of iNOS in TTM-treated post-cardiac arrest patients.

## Limitations

This research is a feasibility study aimed to investigate if RBM3 and CIRP are detectable in human blood samples and if their expressions are regulated by TTM during post-cardiac arrest treatment. Due to the small heterogenous study cohort no conclusion between RBM3 or CIRP expression and their impact on the neurological outcome or further clinical parameters of the patient could be drawn. For this purpose, further research with larger patient numbers is required. In future studies regulation of RBM3 protein should be investigated in full blood samples and not in serum samples as RBM3 primarily function as an intracellular protein and might not be released to the circulatory system. In our study design we did not provide a normothermic control group, as the international guidelines clearly recommend a targeted temperature management between 33 and 36°C for the first 24 hours after CA. Therefore, conclusions about the effect of therapeutic hypothermia on the expression of the inflammatory biomarkers in our patient cohort are limited.

## Conclusion

TTM is used in different clinical settings for neuroprotection, albeit the underlying molecular mechanisms are not fully understood. As indicated by recent experimental data, the cold-inducible proteins, RBM3 and CIRP, are suggested to play a major role in hypothermia-induced neuroprotection. Our feasibility study demonstrates a translation of the experimental findings to the clinic, thus supporting the concept that RBM3 mRNA expression is temperature regulated in a TTM treated post-cardiac arrest cohort and could possibly contribute to the clinical efficacy of therapeutic hypothermia. RBM3 is a feasible biomarker candidate to ensure the effective application of TTM, and its pharmacological induction could be a potential future treatment strategy for neuroprotection.

## Supporting information

S1 Table Raw data of mRNA expressed as Δct and RBM3 protein expressed in pg/ml. (XLSX)

## Abbreviations

CA: Cardiac arrest
CIRP: Cold inducible RNA-binding protein
CPC: cerebral performance category
CPR: cardiopulmonary resuscitation
ICU: intensive care unit
IHCA: in-hospital cardiac arrest
Il-6: Interleukin-6
iNOS: inducible nitrite oxide synthase
MCP-1: monocyte chemotactic protein 1
NSE: neuron specific enolase
OHCA: out-of-hospital cardiac arrest
RBM3: RNA-binding motif 3
ROSC: return of spontaneous circulation
tROSC: time to return of spontaneous circulation
TTM: targeted temperature management
